# Interstitial Lung Disease in 2025—Progress, Challenges, and Hope Ahead

**DOI:** 10.3390/jcm14186673

**Published:** 2025-09-22

**Authors:** Sameep Sehgal, Atul Mehta

**Affiliations:** Department of Pulmonary Medicine, Integrated Hospital Institute, Cleveland Clinic, Cleveland, OH 44195, USA; mehtaa1@ccf.org

Interstitial lung disease (ILD) remains one of the most challenging conditions in respiratory medicine, with outcomes often as poor as those seen in advanced malignancies. Despite a decade of progress in diagnostics and management, ILD continues to be a relentlessly progressive disorder for many patients. Mortality remains high, and the burden on patients, caregivers, and health systems is immense; as such, ILD remains a focus of intensive research and clinical innovation.

The defining feature of ILD is its heterogeneity. The term “ILD” encompasses a spectrum of fibrosing and inflammatory lung disorders, ranging from idiopathic pulmonary fibrosis (IPF) to connective tissue disease-associated ILD, hypersensitivity pneumonitis, and rarer entities such as pleuroparenchymal fibroelastosis. Each subtype presents unique clinical, radiological, and histopathological characteristics, underscoring the need for personalized approaches to diagnosis and care. A “one-size-fits-all” strategy is not adequate; individualized management anchored in disease phenotyping and patients’ personal values should be the standard of care.

Our understanding of ILD has evolved, leading to refinements in nomenclature and diagnostic criteria. The updated 2025 guidelines provide new terminology and frameworks that reflect current knowledge of disease biology and progression [1]. Clinicians now have access to emerging blood-based biomarkers, genomic assays, and advanced imaging tools that may improve early detection and prognostication. While many remain investigational, such tools bring us closer to the use of precision medicine in ILD. These advances also facilitate better stratification in clinical trials, ensuring that novel therapies are tested in the patients most likely to benefit from them. Despite these advances, the role of the multidisciplinary team (MDT) remains central in reaching accurate diagnoses, ensuring nuanced decision-making in a field where subtle distinctions can profoundly influence management [2].

Therapeutically, the last transformative breakthrough in ILD came in 2014 with the introduction of antifibrotic agents, which slowed IPF disease progression for the first time and later demonstrated benefits across a broader spectrum of progressive fibrosing ILD [3,4]. Yet antifibrotics remain imperfect solutions: they mitigate but do not halt fibrosis, and they do not reverse established disease. Encouragingly, the therapeutic pipeline has accelerated. Multiple phase 2 and 3 trials are investigating agents that target novel pathways, including anti-inflammatory, anti-fibrotic, and regenerative strategies. Early data are promising, fueling cautious optimism that the next decade will deliver therapies capable of altering the natural history of these devastating disorders [5]. Nerandomilast, a PDE4B inhibitor, recently reached its primary endpoint in a phase three trial, marking the first positive trial of a novel agent since antifibrotics [6].

This thematic issue of the *Journal of Clinical Medicine* brings together a collection of original research studies and state-of-the-art reviews that reflect both pragmatic aspects of day-to-day ILD care and the frontiers of discovery. Topics range from optimal strategies for symptom management and supportive care to advances in molecular diagnostics and emerging therapeutic modalities. Together, these contributions underscore both the progress achieved and the urgent unmet needs that remain.

As we look ahead, the ILD field stands at a critical juncture. With refined diagnostic tools, deeper biological insights, and a rich therapeutic pipeline, there is reason to hope that patient outcomes will improve in the years to come.
